# Hepatitis B virus genome replication triggers toll-like receptor 3-dependent interferon responses in the absence of hepatitis B surface antigen

**DOI:** 10.1038/srep24865

**Published:** 2016-04-28

**Authors:** Catherine Isabell Real, Mengji Lu, Jia Liu, Xuan Huang, Martin Trippler, Markus Hossbach, Jochen Deckert, Kerstin Jahn-Hofmann, Ludger Markus Ickenstein, Matthias Johannes John, Kathrin Gibbert, Ulf Dittmer, Hans-Peter Vornlocher, Reinhold Schirmbeck, Guido Gerken, Joerg Friedrich Schlaak, Ruth Broering

**Affiliations:** 1 Department of Gastroenterology and Hepatology, University Hospital at the University Duisburg-Essen, Essen, Germany; 2 Institute of Virology, University Hospital at the University Duisburg-Essen, Essen, Germany; 3 Department of Infectious Disease, Union Hospital, Tongji Medical College, Huazhong University of Science and Technology, Wuhan, China; 4 Roche Kulmbach GmbH, Kulmbach, Germany; 5 Axolabs GmbH, Kulmbach, Germany; 6 Sanofi-Aventis Deutschland GmbH, Nucleic Acid Therapeutics Frankfurt, Germany; 7 Boehringer Ingelheim Pharma GmbH Biberach, Biberach an der Riß, Germany; 8 Moderna Therapeutics, Cambridge, Massachusetts, USA; 9 Department of Internal Medicine, University Hospital at the University of Ulm, Ulm, Germany; 10 Evangelisches Klinikum Niederrhein gGmbH, Duisburg, Germany

## Abstract

The hepatitis B virus (HBV) has been described as stealth virus subverting immune responses initially upon infection. Impaired toll-like receptor signaling by the HBV surface antigen (HBsAg) attenuates immune responses to facilitate chronic infection. This implies that HBV replication may trigger host innate immune responses in the absence of HBsAg. Here we tested this hypothesis, using highly replicative transgenic mouse models. An HBV replication-dependent expression of antiviral genes was exclusively induced in HBsAg-deficient mice. These interferon responses attributed to toll-like receptor 3 (TLR3)-activated Kupffer and liver sinusoidal endothelial cells and further controlled the HBV genome replication. However, activation of TLR3 with exogenous ligands indicated additional HBs-independent immune evasion events. Our data demonstrate that in the absence of HBsAg, hepatic HBV replication leads to Tlr3-dependent interferon responses in non-parenchymal liver cells. We hypothesize that HBsAg is a major HBV-mediated evasion mechanism controlling endogenous antiviral responses in the liver. Eradication of HBsAg as a therapeutic goal might facilitate the induction of endogenous antiviral immune responses in patients chronically infected with HBV.

Chronic viral hepatitis caused by hepatitis B virus (HBV) infection is among the most common causes of liver-related morbidity and mortality worldwide^1^. The outcome of HBV infection and its pathogenesis and chronicity are affected by complex interactions between the virus and the immune system. In recent years, it has been shown that the hepatic innate immune system plays an important role in the detection and elimination of hepatotropic pathogens^2^. However, HBV has been described as a stealth virus, with diverse evasion strategies that subverts the innate and adaptive immune systems and leads to failure to induce antiviral immune responses upon infection, as has been shown in HBV-infected chimpanzees^3^,^4^ and humans^5^,^6^,^7^.

Various studies have shown that HBV is highly sensitive to toll-like receptor-induced antiviral mechanisms^8^ mediated by non-parenchymal liver cells (NPCs)^9^. Toll-like receptors are part of the innate immune system; they recognize foreign viral or microbial molecules and therefore play an important role in first-line defense. However, the abrogation of antiviral toll-like receptor signaling by various viral components, such as HBV polymerase^10^, hepatitis B excretory and x antigens (HBeAg, HBxAg)^11^, HBV virions, and hepatitis B surface antigen (HBsAg)^12^, attenuates innate and adaptive immune responses^13^. HBsAg in particular functions as a high-dose tolerogen, blocking local and systemic immune responses. HBsAg is part of the infectious particle, but most HBsAg is secreted as non-infectious filamentous or spherical subviral particles. These subviral particles seem to absorb virus-neutralizing antibodies^14^. High serum levels of HBsAg are associated with an inefficient CD8 T-cell response^15^, affecting the efficiency of adaptive immune mechanisms. Previous findings from our group suggest that Tlr3-activated NPCs can potently suppress HBV replication^9^; however, HBV antagonizes this toll-like receptor signaling by virions, HBeAg, and HBsAg^12^. Jiang *et al.* recently showed that HBsAg inhibits the Tlr3-mediated immune response in NPCs *in vitro*^13^. Aim of the present study was to characterize HBV-mediated antiviral innate immune responses *in vivo*, which were detectable in a highly replicative HBV transgenic mouse model that lacks the small HBsAg. The comparison with an HBsAg-recovered mouse strain should give insights into the role of the HBsAg in immune induction and evasion. One future therapeutic goal might be the complete eradication of HBsAg, thereby facilitating the induction of endogenous antiviral immune responses against HBV.

## Results

### Transgenic HBV-s-mut mice exhibit HBV replication–dependent interferon responses in the liver

**Figure 1:**
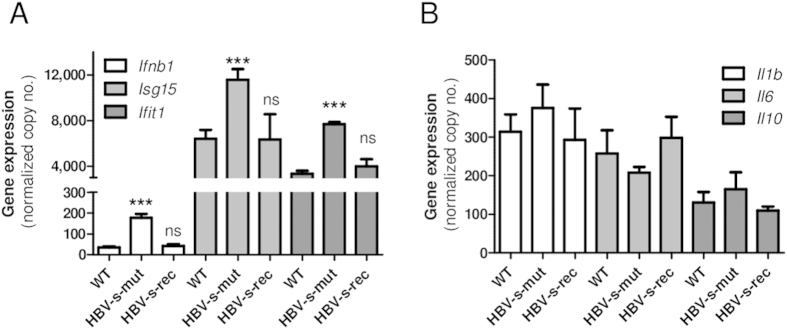
Hepatic interferon responses can be detected in HBV-s-mut but not HBV-s-rec mice. Two-month-old transgenic HBV mice (HBV-s-mut, HBV-s-rec) and wild type littermates (WT) and were put to death, RNA from liver tissue was extracted and changes in gene expression of *Ifnb1*, *Isg15*, *Ifit1* (**A**) and *Il1b*, *Il6*, *Il10* (**B**) were determined by quantitative RT-PCR. Copy numbers were normalized to 100,000 copies of GAPDH (mean values ± SEM). Group size n = 4; ***p < 0.001

HBV transgenic mouse models harboring a 1.4-fold overlength genome lacking (HBV-s-mut) or expressing a functional HBsAg (HBV-s-rec) were initially analyzed for hepatic innate immune signatures. Gene expression of antiviral and inflammatory cytokines was determined by qRT-PCR in two-month-old mice. Compared to wild type littermates, HBV-s-mut mice exhibited elevated expression levels of interferon beta (*Ifnb1*), Isg15 ubiquitin-like modifier (*Isg15*) and *Ifit1* (Fig. 1A), whereas gene expression of Interleukin-1 beta (*Il1b*), *Il6* and *Il10*, was not altered (Fig. 1B). However, the *Ifnb1* expression in the liver did not result in detectable Ifnb1 levels in the serum of these mice, determined by enzyme linked immunosorbent assay (data not shown). Interestingly, the HBV-s-rec mice showed neither an increased expression of inflammatory cytokines nor the induction of an antiviral response (Fig. 1A,B), compared to the wild type littermates. To evaluate the impact of these findings, viral replication and hepatotoxicity were compared between HBV-s-mut and HBV-s-rec mice. Viral replication, indicated by the levels of HBeAg, HBcAg and HBV DNA in liver tissue, did not significantly differ between HBV-s-mut and HBV-s-rec mice. However, HBsAg levels in liver tissue and serum were significantly increased in the HBV-s-rec strain. The de-Ritis-ratio (AST/ALT), an indicator for liver damage, was slightly, but not significantly elevated in the HBV-s-rec strain (Table 1). The major differences between HBV-s-mut and HBV-s-rec strains are the expression and secretion of the HBsAg.

**Figure 2:**
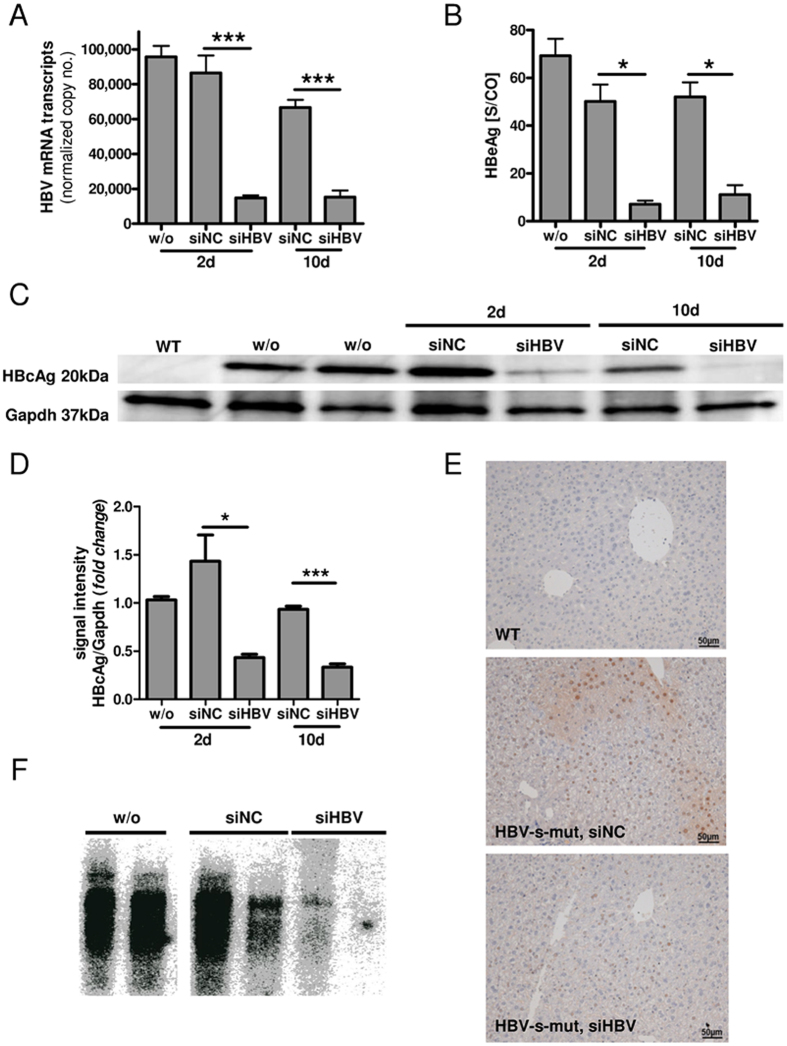
Application of HBV-specific siRNA efficiently suppresses HBV replication *in vivo*. Two-month-old transgenic HBV (HBV-s-mut) mice were given 200 μl of small interfering RNA (siRNA) (4 μg/g body weight) via tail vein injection. Mice were put to death 2 days or 10 days after injection, and liver and blood samples were collected. RNA was extracted from liver tissue, and changes in HBV mRNA expression were determined by quantitative reverse transcription polymerase chain reaction (qRT-PCR) (**A**). Copy numbers were normalized to 100,000 copies of glyceraldehyde 3-phosphate dehydrogenase (*Gapdh*) (mean ± SEM). Serum was prepared, and hepatitis B excretory antigen (HBeAg) levels were measured by chemiluminescent microparticle immunoassay (CMIA) (mean ± SEM) (**B**). Proteins were extracted from liver tissue, and HBcAg was detected by western blot analysis (**C**). Densitometric analysis was performed (**D**). Liver tissue was fixed, embedded in paraffin, and sectioned; it was then stained with HBcAg/hematoxylin. Images (20x optical magnification) show cells from wild type (WT) mice, from HBV-s-mut mice given non-template control siRNA (siNC), and from HBV-s-mut mice given HBxAg-targeting siRNA (siHBV) (**E**). DNA was extracted from liver tissue, and changes in DNA replication of HBV were determined by Southern blot analysis (**F**). Group size n = 3 (**A**,**C/D**) or exemplarily n = 2 (**B**,**F**) animals; *p < 0.05, ***p < 0.001; d, day; w/o, without treatment.

**Figure 3:**
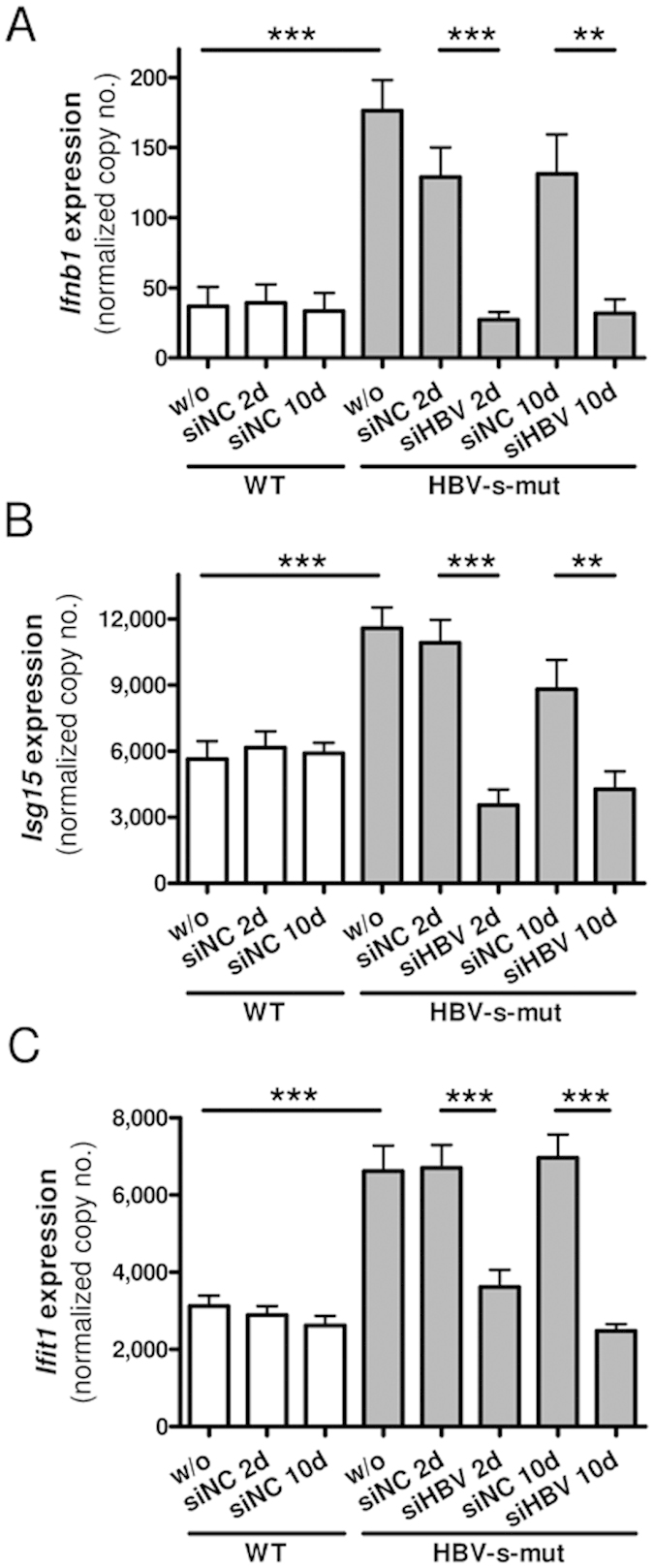
HBsAg-deficient transgenic HBV mice exhibit HBV replication-dependent interferon responses. Two-month-old transgenic HBV (HBV-s-mut) mice or HBV-negative littermates (WT) were given 200 μl of small interfering RNA (siRNA) (4 μg/g body weight) via tail vein injection. Mice were put to death 0, 2, or 10 days after injection. RNA was extracted from liver tissue, and changes in gene expression of *Ifnb1* (**A**), *Isg15* (**B**), and *Ifit1* (**C**) were determined by quantitative reverse transcription polymerase chain reaction (qRT-PCR). Copy numbers were normalized to 100,000 copies *Gapdh* (mean ± SEM). Group size n = 4 animals; **p < 0.01, ***p < 0.001; d, day; siHBV, HBxAg-targeting siRNA; siNC, non-template control siRNA; HBV-s-mut, Tg1.4HBV-S-Mut3; w/o, without treatment; WT, wild type.

To further analyze the hepatic interferon response in HBV-s-mut mice, HBV-targeting, LNP-formulated, 2′O-methyl–modified siRNAs (siHBV) were used as a potent tool for suppressing HBV in hepatocytes without inducing antiviral off-target responses^16^,^17^. Either siHBV or siNC were injected i.v. (4 μg/g body weight), the animals were put to death after 2 or 10 days, and hepatic gene expression was determined by qRT-PCR^18^. The administration of siHBV led to hepatic suppression of HBV mRNA transcripts at day 2 with an efficiency of 82.8% ± 2.3% (mean ± SEM, p = 0.0009). HBV mRNA levels were consistently suppressed for at least 10 days, with a sustained suppression of 77.1% ± 8.2% (p < 0.0001) (Fig. 2A). The HBeAg serum levels were also suppressed by siHBV treatment, reaching 87.2% ± 21.3% (p = 0.02) and 80.0% ± 35.5% (p = 0.02) suppression at day 2 and day 10 after treatment, respectively (Fig. 2B). In addition, hepatic HBcAg levels were reduced after siHBV treatment, as determined by western blot analysis (Fig. 2C,D). Furthermore, immunohistochemical staining of liver tissues revealed a nuclear as well as cytosolic distribution of HBcAg, which was highly reduced after siHBV treatment (Fig. 2E). Southern blot analysis demonstrated a reduction of the hepatic HBV DNA level after siHBV treatment (Fig. 2F). Interestingly, siHBV-mediated suppression of viral replication was accompanied by the normalization of *Ifnb1*, *Isg15*, and *Ifit1* gene expression. Thus, interferon and interferon-stimulated gene expression levels were reduced to basal expression levels at day 2 and day 10 after treatment with siHBV (Fig. 3A–C). As the hepatic expression levels of these genes did not differ between wild type and HBV-s-rec mice (Fig. 1A), wild type animals were chosen as control group, especially to indicate that application of modified siRNA (here siNC) did not lead to off target immune induction. These results led us to hypothesize that the interferon responses observed in HBV-s-mut mice may be dependent upon an active HBV replication process.

### HBV replication-dependent interferon responses are mediated by KCs and LSECs

To analyze the origin of the hepatic interferon response, which was exclusively shown in HBV-s-mut mice, PMHs and a mix of NPCs were isolated, including KCs, LSECs and to lesser extent hepatic stellate cells, T cells or natural killer cells. RNA was extracted to determine the gene expression of *Ifnb1*, *Isg15*, and *Ifit1* by qRT-PCR. As shown in supporting information, the interferon responses were restricted to the NPC fraction. Comparison of gene expression levels in NPCs demonstrated that *Ifnb1* expression was 5.4 ± 1.9-fold higher (p < 0.01) (Fig. SI 1A), induction of *Isg15* was 5.5 ± 1.7-fold higher (p = 0.02) (Fig. SI 1B), and expression of *Ifit1* was 3.1 ± 0.7-fold higher (p < 0.01) (Fig. SI 1C) in HBV-s-mut-derived NPCs than in those obtained from control mice (wild type), which showed basal expression of these genes. Furthermore, PMHs isolated from HBV-s-mut animals did not significantly respond to either HBV replication or the endogenous Ifnb1 expressed by the neighboring NPCs.

**Figure 4:**
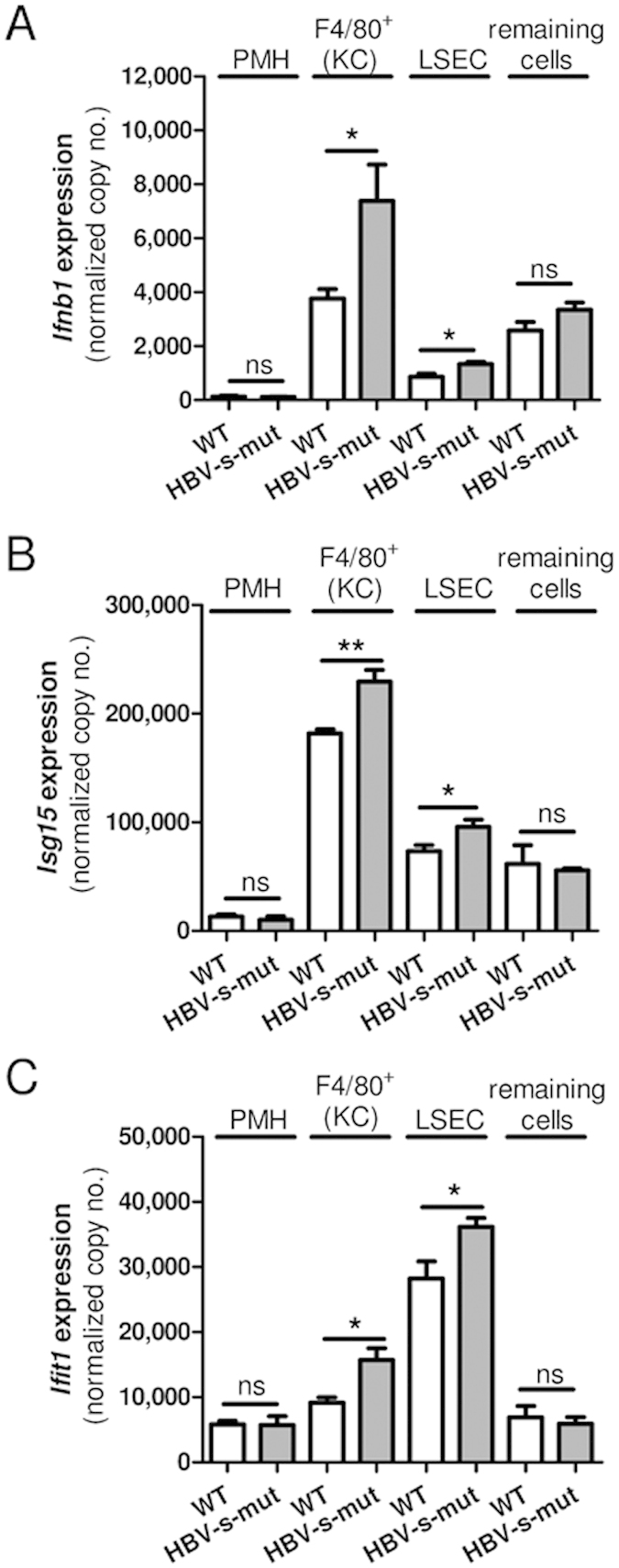
HBV-induced interferon responses are mediated by F4/80+ cells (KCs) and liver sinusoidal endothelial cells (LSECs). Primary murine hepatocytes (PMH), KCs, LSECs and a remaining cell fraction were isolated from two-month-old transgenic HBV (HBV-s-mut) mice and from HBV-negative littermates (WT). RNA was extracted; gene expression of *Ifnb1* (**A**), *Isg15* (**B**), and *Ifit1* (**C**) was determined by quantitative reverse transcription polymerase chain reaction (qRT-PCR). Copy numbers were normalized to 100,000 copies of *Gapdh* (mean ± SEM). Group size n = 4 animals; *p < 0.05, **p < 0.01, ***p < 0.001; ns, not significant.

To get an idea which of the non-parenchymal liver cell types were mediating the HBV replication-dependent interferon response, we further separated F4/80^+^ cells (KCs), LSECs and a fraction of remaining cells, including hepatic stellate cells, T cells and natural killer cells. Comparison of gene expression levels demonstrated that *Ifnb1* expression reached maximum expression level in HBV-s-mut-derived KCs (7,372 ± 1,358 normalized copy no., mean ± SEM). In the correspondent LSECs *Ifnb1* expression was also significantly induced (1,328 ± 77.2 normalized copy no., mean ± SEM) (Fig. 4A). In contrast, *Ifnb1* expression in liver tissue of HBV-s-mut mice reached a level of 176.4 ± 21.6 normalized copy no. (mean ± SEM; Fig. 3A). This might be due to the fact, that PMH, which represent the largest liver cell population, only showed marginal *Ifnb1* expression (109.4 ± 19.5 mean ± SEM; Fig. 4A). The *Isg15* and *Ifit1* gene expression also reached maximum expression levels in KCs (*Isg15*: 229,466 ± 10,647 normalized copy no., mean ± SEM) and LSECs (*Ifit1*: 36,128 ± 1,394 normalized copy no., mean ± SEM), respectively (Fig. 4B,C). However, PMHs and the remaining cell fraction isolated from HBV-s-mut mice did not show an elevated expression of these genes. Comparison had been made to wild type littermates, which showed the same hepatic gene expression pattern as HBV-s-rec mice. These results demonstrated that mainly KCs and to a lesser extent LSECs are responsible for the interferon response detected in the liver of HBV-s-mut mice.

### Interferon responses in HBV-s-mut mice are mediated by Toll-like receptor 3 activation and they limit HBV replication

**Figure 5:**
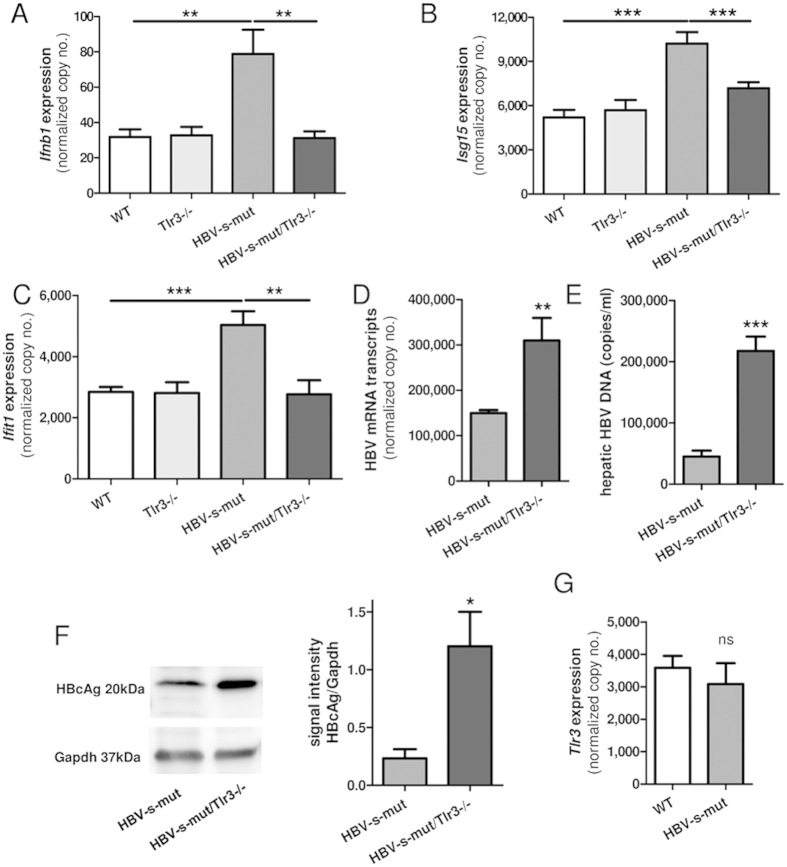
Tlr3 is consistently activated in transgenic HBV-s-mut mice and thereby controls HBV replication. Transgenic HBV (HBV-s-mut) mice were crossbred with Toll-like receptor 3-deficient (Tlr3−/−) mice. Two-month-old HBV-negative littermates (WT), Tlr3−/− mice, HBV-s-mut mice, and HBV-s-mut/Tlr3−/− mice were put to death. RNA was extracted from liver tissue and changes in gene expression of *Ifnb1* (**A**), *Isg15* (**B**), *Ifit1* (**C**), *Tlr3* (**G**) and HBV mRNA expression (**D**) were determined by quantitative reverse transcription polymerase chain reaction (qRT-PCR). Copy numbers were normalized to 100,000 copies of *Gapdh* (mean ± SEM). DNA was extracted from liver tissue, and changes in HBV DNA were quantified by PCR (**E**). Proteins were extracted from liver tissue, HBcAg was detected by western blot analysis and densitometric analysis was performed (**F**). Group size n = 8 (**A**–**D**), n = 4 (**E**,**G**) and n = 3 (**F**) animals; *p < 0.05, **p < 0.01, ***p < 0.001; HBV-s-mut, Tg1.4HBV-S-Mut3; WT, wild type.

The previously reported HBV-mediated expression of *Ifnb1* led us to question whether the immune response was mediated by Tlr3. To investigate this hypothesis, HBV-s-mut mice were crossbred with Tlr3−/− knockout animals and then qRT-PCR was used to determine hepatic interferon responses. Interestingly, the interferon and interferon-stimulated gene expression profile showed normal levels in HBV-s-mut/Tlr3−/− mice, and reached expression levels as shown for WT or Tlr3−/− control animals. Thus, the interferon and interferon-stimulated gene expression levels, which were enhanced in HBV-s-mut mice (2.5 ± 0.6-fold induction of *Ifnb1*, p < 0.0029; 2.0 ± 0.2-fold induction of *Isg15*, p < 0.001; and 1.8 ± 0.2-fold induction of *Ifit1*, p < 0.0001) (Fig. 5A–C), were at basal levels in HBV-s-mut/Tlr3−/− mice. These findings indicate that the HBV-induced interferon response depended on a functional Tlr3 system. Interestingly, HBV replication was enhanced in Tlr3-deficient HBV-s-mut animals, as indicated by increased HBV mRNA expression levels (2.0 ± 0.9-fold induction, p < 0.01) (Fig. 5D), hepatic HBV DNA levels (4.8 ± 0.5-fold induction, p = 0.0005) (Fig. 5E), and HBcAg protein (5.2 ± 1.1-fold induction, p = 0.03) determined by quantitative PCR and western blot analysis (Fig. 5F), respectively. However, the hepatic expression of *Tlr3* itself did not differ between HBV-s-mut mice and WT mice (Fig. 5G). These findings may suggest that the continuous activation of Tlr3 in HBV-s-mut animals can control the level of HBV replication.

### Exogenous activation of Tlr3 by Poly(I:C) in HBV-s-mut mice is suppressed *in vivo* and *in vitro*

**Figure 6:**
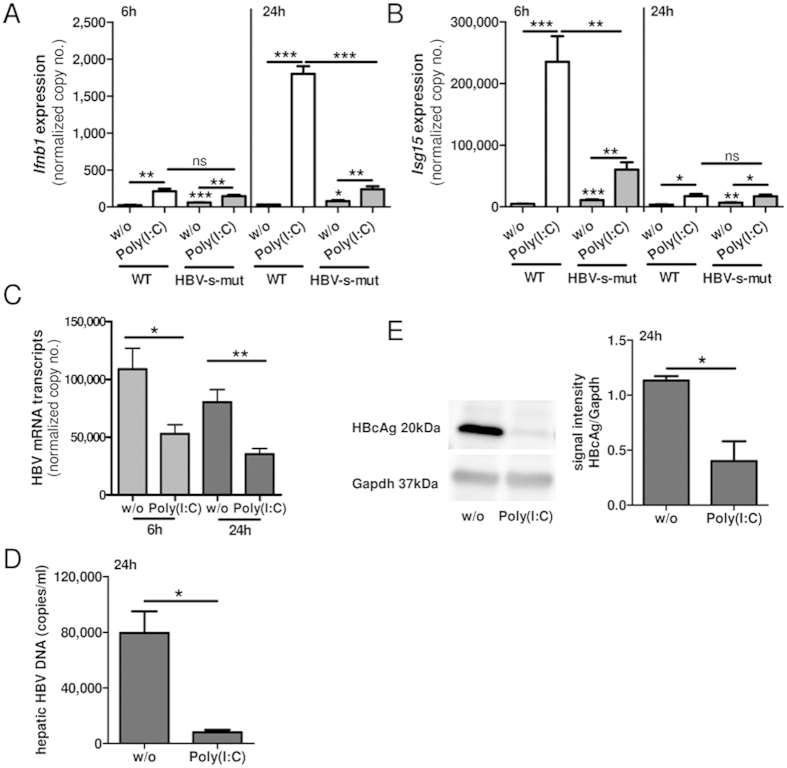
Activation of TLR3 by Poly(I:C) in HBV-s-mut mice is suppressed but still effective *in vivo.* Two-month-old male transgenic HBV (HBV-s-mut) mice and their wild type (WT) littermates were given Toll-like receptor 3 (Tlr3) ligand polyinosinic-polycytidylic acid (Poly[I:C]; 4 μg/g body weight) via tail vein injection. Mice were put to death 6 h or 24 h after injection. RNA was extracted, and changes in gene expression of *Ifnb1* (**A**), *Isg15* (**B**), and HBV (**C**) were determined by quantitative reverse transcription polymerase chain reaction (qRT-PCR). Copy numbers were normalized to 100,000 copies of *Gapdh* (mean ± SEM). DNA was extracted from liver tissue, and changes in HBV DNA were quantified by PCR (**D**). Proteins were extracted from liver tissue, HBcAg was detected by western blot analysis and densitometric analysis was performed (**E**). Group size n = 3 animals; *p < 0.05, **p < 0.01, ***p < 0.001; HBV-s-mut, Tg1.4HBV-S-Mut3; w/o, without treatment.

To determine whether Tlr3 activation in HBV-s-mut mice is controlled by additional HBV evasion strategies, Tlr3 ligand Poly(I:C) was administered (4 μg/g body weight) i.v. to HBV-s-mut mice and WT littermates. Mice were put to death 6 or 24 h after injection. RNA was extracted from liver tissue, and changes in gene expression of *Ifnb1*, *Isg15*, and HBV RNA were determined by qRT-PCR. Although the gene expression of interferons and interferon-stimulated genes was continuously elevated in HBV-s-mut mice, Tlr3 ligands further induced the expression of *Ifnb1* (3.1 ± 0.8-fold change in HBV-s-mut mice). However, 24 h after Poly(I:C) injection the induction of *Ifnb1* expression was much higher in WT littermates (57.5 ± 4.4-fold induction, p < 0.0001). Poly(I:C)-induced expression of *Ifnb1* was 86.7% ± 3.2% (p < 0.0001) lower in HBV-s-mut mice than in WT littermates (Fig. 6A). Poly(I:C) administration also induced *Isg15* expression in both HBV-s-mut mice (5.6 ± 1.6-fold induction, p = 0.0022) and WT mice (51.1 ± 12.7-fold induction, p = 0.0002). Interestingly, this induction reached a peak 6 h after Poly(I:C) injection and was 74.4% ± 7.2% (p = 0.0023) lower in HBV-s-mut mice than in their Poly(I:C)-treated WT littermates (Fig. 6B). This suppressed activation of Tlr3 by Poly(I:C) in HBV-s-mut mice led to suppression of the HBV mRNA levels 6 h (51.5% ± 10.2%, p = 0.00171) and 24 h after injection (55.9% ± 8.4%, p = 0.0036) (Fig. 6C). Hepatic HBV DNA levels (Fig. 6D) and hepatic HBcAg levels (Fig. 6E) were significantly lowered 24 h after Poly(I:C) administration (DNA 89.8% ± 2.0, p = 0.05; HBcAg 64.7% ± 13.9, p = 0.02), determined by quantitative PCR and western blot analysis, respectively. These findings suggest that activation of Tlr3 by exogenous ligands may be controlled by HBV proteins independently of HBsAg. This suggestion is supported by the lack of an endogenous interferon response in the PMH fraction of HBV-s-mut mice, shown in Fig. S1 and Fig. 4.

**Figure 7:**
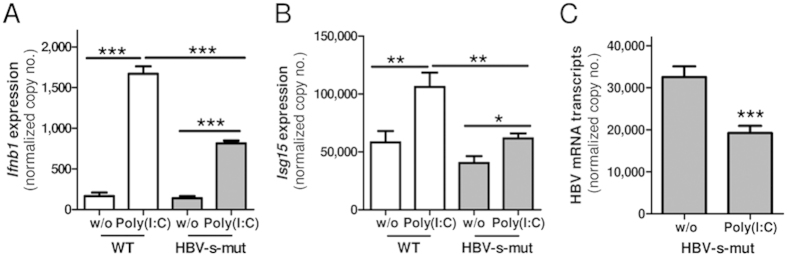
Activation of TLR3 by Poly(I:C) in HBV-s-mut-derived primary hepatocytes is suppressed but still effective. Primary murine hepatocytes (PMHs) were isolated from two-month-old male HBV-s-mut mice and their wild type littermates (WT). The PMHs were isolated, cultured, and stimulated with 25 μg/ml of Tlr3 ligand Poly(I:C) for 6 h. RNA was extracted, and changes in gene expression of *Ifnb1* (**A**), *Isg15* (**B**), and HBV (**C**) were determined by quantitative reverse transcription polymerase chain reaction (qRT-PCR). Copy numbers were normalized to 100,000 copies of *Gapdh* (mean ± SEM). Group size n = 3 animals; *p < 0.05, **p < 0.01, ***p < 0.001; HBV-s-mut, Tg1.4HBV-S-Mut3; w/o, without treatment.

Furthermore, we validated the *in vivo* findings by performing *in vitro* studies using PMHs isolated from two-month-old male HBV-s-mut mice and their WT littermates. These cells were cultured and stimulated with Poly(I:C) (25 μg/ml) for 6 h. RNA was extracted, and changes in the expression of *Ifnb1*, *Isg15*, and HBV RNA were determined by qRT-PCR. Poly(I:C) administration significantly induced *Ifnb1* expression in PMHs isolated from WT mice (10.1 ± 0.9-fold induction, p < 0.001) and HBV-s-mut animals (5.9 ± 0.4-fold induction, p < 0.001); however, *Ifnb1* expression was significantly suppressed (41.8% ± 6.1%, p < 0.001) in PMHs from HBV-positive animals (Fig. 7A). In PMHs from WT mice, Poly(I:C) stimulation induced a 1.8 ± 0.4-fold higher expression of *Isg15* (p < 0.01). This phenomenon was also observed in PMHs from HBV-s-mut mice, which showed a 1.5 ± 0.2-fold induction (p = 0.02). However, this response was reduced by 51.3% ± 3.6% (p < 0.01) (Fig. 7B). Nevertheless, Tlr3 activation in PMHs derived from HBV-s-mut mice led to 40.8% ± 8.5% (p < 0.001) suppression of HBV mRNA expression (Fig. 7C). These results indicate that *in vitro* stimulation of Tlr3 was also suppressed but not abrogated in HBV-s-mut-derived PMHs.

## Discussion

To date mechanisms of immune responses against HBV still remained unclear. To gain insight into the host-virus interaction suitable animal models need to be developed and analyzed^18^. It has been shown that significant induction of interferons is lacking in HBV-infected patients^19^ and chimpanzees soon after infection^3^. Therefore, HBV has been called a stealth virus that subverts the host’s immune system^3^,^4^. In the present study we investigated the consequences of HBV replication on the innate immunity by characterizing hepatic immune responses in HBV transgenic mice. HBV replication solely induced antiviral responses in the absence of HBsAg. Transgenic HBV-s-mut mice exhibited a continuous, replication-dependent interferon response that was mediated by Tlr3-activated NPCs, in detail by activated KCs and LSECs. Putative HBV-derived ligands for Tlr3 are secondary structures of full-length or truncated RNAs, detectable in the serum of HBV-infected patients^20^,^21^. Furthermore, HBV RNA containing cell debris might also be the source of the Tlr3 ligand, in spite of the fact that no macroscopic or microscopic cell injury could be found in HBV-s-mut animals. We hypothesize that serum HBsAg prevents endogenous interferon responses, thereby supporting the stealth virus characteristics of HBV. It has been recently shown that HBsAg potently attenuates TLR3-induced cytokine expression and IRF-3 activity in murine KCs and LSECs, *in vitro*^13^. It is supposed, that IL10 play a role^13^, however the underling mechanisms remained unclear.

Currently, there is no efficient cure for chronic HBV infection. Novel antiviral agents suppress HBV replication very efficiently but fail to eliminate the covalently closed circular DNA (cccDNA)^22^, which represents back-up copies of the virus. Suggested clearance mechanisms are apoptosis of infected hepatocytes and degradation of the nuclear HBV cccDNA by the non-cytolytic mechanism^23^ mediated by IFNG^24^,^25^, Il18^26^, IL12^27^,^28^, IL6^29^, and TNFA^30^,^31^. In this process adaptive immune responses play an important role, but functional and efficient innate immune responses are also necessary because they in turn regulate the adaptive responses to HBV^32^. The findings of the present study indicate that HBV-s-mut mice, which lack HBsAg, exhibited continuous hepatic induction of antiviral genes, thereby controlling viral replication. The induction of these antiviral genes could not be observed in the HBV-s-rec mice, which showed recovered expression and secretion of the HBsAg. These results suggest that production and secretion of HBsAg can effectively inhibit the endogenous induction of antiviral immune responses in the hepatic environment. These findings correlate with those from clinical studies, which showed that patients with high HBs-antigenemia exhibit suppression of TLR-induced interferon expression^13^. Other studies have also shown that, in liver tissue and peripheral blood mononuclear cells from patients with chronic hepatitis B (CHB), TLR3 expression itself is substantially reduced. Thus IFNB1 production is also suppressed in CHB patients^5^,^33^. Impairments in the expression and signaling of TLR3 result in an inappropriate antiviral innate immune response in HBV-infected patients^34^. Accordingly, abrogation of HBV-induced interferon responses was observed in Tlr3-deficient HBV-s-mut mice, as shown in the current study, indicating the involvement of Tlr3 in antiviral responses against HBV. Interestingly, the absence of Tlr3 in this system provoked an increase in the hepatic levels of HBV mRNA, HBV DNA and HBcAg, indicating an enhanced HBV replication in these mice. Although HBV-s-mut mice-derived PMHs did not show significant induction of *Ifnb1* or interferon stimulated gene expression, in liver tissue viral replication was controlled by constitutive Tlr3 activation. The NPCs continuously produced antiviral mediators, which limited viral replication. These contradictory findings might be explained by either local restrictions of antiviral immune induction and immune tolerance or indirect action of these antiviral mediators. We here conclude that therapeutic suppression of HBsAg in CHB patients may overcome immune evasion by rebooting the antiviral innate immune responses. This hypothesis needs to be proven in future studies, where neutralization of serum HBsAg in the HBV-s-rec model might reactivate endogenous immune responses.

It has been suggested that the inhibition of innate immunity by HBV may not be very robust, because it can be overcome by exogenous stimulation, for example with toll-like receptor ligands^8^,^35^. Several *in vivo* and *in vitro* models of HBV infection have shown that the inhibition of HBV replication by toll-like receptor ligands (Tlr3/4/5/7/9) is dependent on type I Interferons^8^,^9^. These findings are confirmed by the results of the current study; the activation of Tlr3 by its ligand Poly(I:C) led to significant suppression of HBV replication, both *in vivo* and *in vitro*. The administration of Poly(I:C) to transgenic HBV-s-mut mice demonstrated an inducible and functionally active Tlr3 signaling pathway that produces effective but partially suppressed interferon responses. The reason for this partial suppression may be that, in addition to HBsAg, which is absent from the system used in this study, various other proteins of the hepatitis B virus are known to block the toll-like receptor system. For example, overexpression of HBV polymerase in a hepatoma cell line (HepG2/HepG2.215) blocks downstream signals of the pathogen recognition receptors TLR3 and retinoic acid–inducible gene 1 (RIG-I). It has been shown that HBV polymerase also inhibits TANK-binding kinase 1/IkappaB kinase epsilon (IKKε) activity by disrupting the interaction between IKKε and DEAD box helicase 3 , thereby suppressing the signaling of interferon regulatory factor 3 (IRF3) and IRF7^10^. Furthermore, HBxAg has been shown to suppress IFNB1 production in a HepG2 cell line. HBxAg promotes the degradation of mitochondrial antiviral-signaling protein, a downstream adaptor molecule in the RIG-I pathway, by ubiquitinylation, thereby preventing the induction of IFNB1^11^. However, HBsAg has been shown to potently inhibit Tlr3-induced activation of Irf3, nuclear factor kappa B, and mitogen-activated protein kinases in murine NPCs^9^,^13^. In the current study we showed that the immune evasion observed in Tg1.4HBV-S-Mut3 was not strong enough to completely block endogenous antiviral responses. This finding suggests that HBsAg might be a major effector mediating the blockade of antiviral responses observed in HBV-s-rec mice, HBV-infected chimpanzees^3^ or patients with chronic hepatitis B^5^,^6^,^7^. In the analyzed HBV-s-mut model, HBV replication-dependent interferon responses were mediated by the activation of KCs and LSECs, not by HBV-replicating hepatocytes, which did not respond even to endogenous *Ifnb1*. This interferon response was not observed in the HBsAg-recovered strain, supporting the hypothesis that HBsAg is primarily responsible for HBV evasion strategies *in vivo.* However, exogenous application of Tlr3 ligand led to suppression of HBV replication in the present study, confirming Tlr3-mediated antiviral responses observed in a distinct HBV transgenic mouse model as well as in the HBV hydrodynamic injection model^8^,^36^.

The present study demonstrates that HBV replication is able to induce Tlr3-mediated interferon responses in KCs and LSECs, which control hepatic HBV replication in transgenic mice that lack HBsAg. This antiviral response was completely missing in the HBsAg-recovered strain. We therefore hypothesize that HBsAg functions as a high-dose tolerogen that efficiently blocks innate and, subsequently, adaptive immune responses by suppressing the elimination of infected cells by the immune system^4^,^15^,^37^. This led us to hypothesize that patients might be able to mount an efficient antiviral immune response against HBV once the HBsAg could be eliminated from the blood circulation.

## Methods

### Materials

The small interfering RNAs (siRNAs) formulated with lipid nanoparticles (LNPs) that were used for *in vivo* studies were provided by Axolabs GmbH (formerly Roche Kulmbach GmbH, Kulmbach, Germany). The HBV-targeting siRNA which binds to the 3′-region of all viral transcripts (siHBV, target sequence, 5′-ACCUCUGCCUAAUCAUCUC-3′) and the non-template control (siNC, target sequence, 5′-CUUACGCUGAGUACUUCGA-3′) were 2′O-methyl–modified at the ribose backbone to prevent nuclease-driven degradation and to minimize siRNA-mediated off-target immune induction^16^,^38^. Polyinosinic-polycytidylic acid (Poly[I:C]) was obtained from Invivogen (Toulouse, France).

### Animals

Tg1.4HBV-S-Mut3 mice harbor a single mutation; the translational start codon of the small HBsAg gene (SHBs) is mutated from ATG to ACG, resulting in the absence of SHBs expression^39^. These mice produce no infectious or subviral particles. The Tg[Alb-1,HBV]Bri66 strain^40^, genetically engineered by Chisari *et al.* highly express all types of HBsAg. Crossbreeding of these two strains has been successfully performed to generate HBV transgenic mice, expressing all HBV gene products^39^. Propagation of Tg1.4HBV-S-Mut3 or Tg[Alb-1,HBV]Bri66 was done by breeding with C57BL/6J mice. In the present study, the cross bred strain (Tg1.4HBV-S-Mut3 × Tg[Alb-1,HBV]Bri66) is designated HBV-s-recover (HBV-s-rec). The Tg1.4HBV-S-Mut3 mice are called HBV-s-mut mice, and their HBV-negative littermates are described as wild type (WT) mice. All enumerated mice as well as the Tlr3−/− knockout mice^41^ were bred at the University Hospital of Essen, fed ad libidum and received humane care according to the criteria outlined in the *Guide for the Care and Use of Laboratory Animals* prepared by the National Academy of Sciences and published by the National Institutes of Health. The animal study design was approved by the local committee (*Landesamt für Natur, Umwelt und Verbraucherschutz*).

### Isolation and culture of murine hepatocytes and NPCs

Primary murine hepatocytes (PMHs), were prepared by liberase perfusion as described previously^12^. PMHs had been separated by low-speed centrifugation steps (50 × g for 10 min at 4 °C). The supernatant containing the non-parenchymal liver cells (NPCs) was centrifuged at 300 × g for 10 min at 4 °C. NPCs were either directly lysed for RNA extraction or Kupffer cells (KCs) and liver sinusoidal endothelial cells (LSECs) were further separated using F4/80- and CD146-directed MicroBeads (Miltenyi Biotec, Bergisch Gladbach, Germany), respectively. KCs and LSECs were lysed for RNA extraction and quantitative reverse transcription polymerase chain reaction (qRT-PCR). PMHs were directly lysed or seeded into collagen I–coated culture plates for 24 h and stimulated with Poly(I:C) (25 μg/ml) for additional 6 h, followed by RNA isolation and qRT-PCR.

### Synthesis of siRNAs, manufacturing and characterization of LNPs

The methods used for synthesizing siRNA and generating LNP formulations are described in Broering *et al.*^16^.

### RNA isolation and quantitative RT-PCR

Total RNA was isolated and purified with Qiazol™ solution (Qiagen, Hilden, Germany) and the RNeasy Mini Kit (Qiagen) according to the manufacturer’s instructions. Quantitative RT-PCR was performed with the QuantiFast SYBR Green RT-PCR Kit (Qiagen) using 0.1 to 0.3 μg of total RNA. HBV mRNA transcripts were analyzed with the sense primer 5′-CCGTCTGTGCCTTCTCATCT-3′ (position nt1549, EcoR1 0/3182) and the antisense primer 5′-TAATCTCCTCCCCCAACTCC-3′ (position nt1755, EcoR1 0/3182), allowing the detection of the diverse HBV mRNAs. For interferon-induced protein with tetratricopeptide repeats 1 (*Ifit1*) the sense primer 5-CTGAAATGCCAAGTAGCAAGG-3′ and antisense primer 5′-CCAAAGGCACAGACATAAGGA-3′ were used. Expression of all other genes was detected by commercially available primer sets (QuantiTec Primer Assay, Qiagen; sequences are not given by the manufacturer). The calculated copy numbers were normalized to the housekeeping gene glyceraldehyde 3-phosphate dehydrogenase (*Gapdh*) which was detected with the sense primer 5′-AAATTCAACGGCACAGTCAA-3′ and the antisense primer 5′-TCTCCATGGTGGTGAAGACA-3′. The RNA analysis was performed considering MIQE guidelines^42^.

### Western blot analysis

Western blot analysis was performed as previously described^43^. The following antibodies were used: anti-HBV core (HBcAg) (16), HBsAg antibody (Aviva Systems Biology, San Diego, CA, USA) and Gapdh (Cell Signaling, Danvers, MA, USA). Signal intensities were measured with FUSION FX7 Advanced system and FUSION-CAPT Advanced software (Vilber Lourmat, Eberhardzell, Germany).

### Detection of HBV DNA

Hepatic HBV DNA was extracted with the QIAamp DNA Mini kit (Qiagen) according to the manufacturer’s instructions. Southern blot analysis was performed as previously described^44^. Hepatic HBV DNA was quantified by PCR using the Platinum SYBR Green Kit (Invitrogen/Life technologies, Darmstadt, Germany) with the sense primer 5′-TGC CTC ATC TTC TTR TTG GTT CT-3′ and the antisense primer 5′-CCC CAA WAC CAK ATC ATC CAT ATA-3′. The primer contained wobble bases (r = purine (A,G), w = weak binding (A,T) and k = keto (G,T)).

### Analysis of serum transaminases, HBeAg and HBsAg

Serum analyses of alanine aminotransferase (ALT) and aspartate aminotransferase (AST) were performed with the Spotchem™II analyzer and Spotchem™II reagent strips (liver-panel1; Arkray, Kyozo, Japan) according to the manufacturer’s instructions. HBeAg and HBsAg were detected in serum samples of HBV transgenic mice with the chemiluminescent microparticle immunoassay (CMIA) ARCHITECT HBeAg and HBsAG assay with the ARCHITECT immunoassay analyzer (Abbott Diagnostics, Wiesbaden, Germany), according to the manufacturer’s instructions.

### Immunohistochemistry of liver tissue

Murine liver tissue samples were fixed in 4.5% Histofix (Carl-Roth, Karlsruhe, Germany) and embedded in paraffin. Sections were cut for immunohistochemistry staining with rabbit polyclonal anti-HBV core antibody (Dako, Hamburg, Germany) and hematoxylin (Carl-Roth, Karlsruhe, Germany). Histologic findings were visualized with an Axioplan microscope and AxioCam Hrc (Carl Zeiss, Jena, Germany).

### Statistical analysis

Data are expressed as mean ± SEM (standard error of mean). Differences between two groups were determined with the Wilcoxon test. Statistical significance was set at the level of p < 0.05.

## Additional Information

**How to cite this article**: Real, C. I. *et al.* Hepatitis B virus genome replication triggers toll-like receptor 3-dependent interferon responses in the absence of hepatitis B surface antigen. *Sci. Rep.* **6**, 24865; doi: 10.1038/srep24865 (2016).

## Supplementary Material

Supplementary Information

## Figures and Tables

**Table 1:** Characteristics of HBV replication in HBVs-mut and HBV-s-rec strains.

	HBV-s-mut (n = 4)	HBV-s-rec (n = 4)	p-value
	(mean ± SEM)	(mean ± SEM)	
HBsAg _SERUM_ [S/CO]	**2.0 ± 0.0**	**226.2 ± 16.2**	**0.0008**
DNA _LIVER_ (copy no./μg DNA)	7,802 ± 2,615	4,722 ± 2,328	0.5176
HBeAg _SERUM_ [S/CO]	56.2 ± 15.6	43.4 ± 9.0	0.5064
HBcAg _LIVER_ (signal intensity HBcAg/Gapdh)	0.84 ± 0.13	1.1 ± 0.1	0.0842
large HBsAg _LIVER_ (signal intensity HBsAg/Gapdh)	**3.8 ± 1.1**	**28.3 ± 3.0**	**0.0002**
middle HBsAg _LIVER_ (signal intensity HBsAg/Gapdh)	**3.0 ± 0.8**	**23.7 ± 1.9**	**<0.0001**
small HBsAg _LIVER_ (signal intensity HBsAg/Gapdh)	**3.4 ± 0.5**	**29.8 ± 1.5**	**0.0013**
De-Ritis-Ratio (AST/ALT)	1.5 ± 0.2	2.8 ± 0.7	0.1025

Footnote: Two-month-old transgenic HBV mice were put to death, liver and serum samples were collected. DNA was extracted from liver tissue, and changes in DNA replication of HBV were determined by quantitative PCR (copy no./μg DNA). Hepatitis B excretory antigen (HBeAg) and Hepatitis B surface antigen (HBsAg) levels in the serum were measured by chemiluminescent microparticle immunoassay (CMIA, [S/CO] = sample relative light units (RLU) per cutoff). Proteins were extracted from liver tissue, and HBV core (HBcAg) or HBsAg were detected by Western blot analysis. Densitometric analysis was performed. Serum parameter (alanine aminotransferase (ALT) and aspartate aminotransferase (AST)) were determined with the Spotchem™II analyzer. Significant changes are given in bold.
