# Supplementary material for: Hepatitis B virus genome replication triggers toll-like receptor 3-dependent interferon responses in the absence of hepatitis B surface antigen
Source: Sci Rep. 2016 Apr 28;6:24865. doi: 10.1038/srep24865 (PMC4848479; doi:10.1038/srep24865)
Supplement: Supplementary Information [file srep24865-s1.pdf]

Supplementary information

Title: Hepatitis B virus genome replication triggers toll-like receptor 3-dependent interferon responses in the absence of hepatitis B surface antigen

Authors: Catherine Isabell Real, Mengji Lu, Jia Liu, Xuan Huang, Martin Trippler, Markus Hossbach, Jochen Deckert, Kerstin Jahn-Hofmann, Ludger Markus Ickenstein, Matthias Johannes John, Kathrin Gibbert, Ulf Dittmer, Hans-Peter Vornlocher, Reinhold Schirmbeck, Guido Gerken, Joerg Friedrich Schlaak, Ruth Broering.

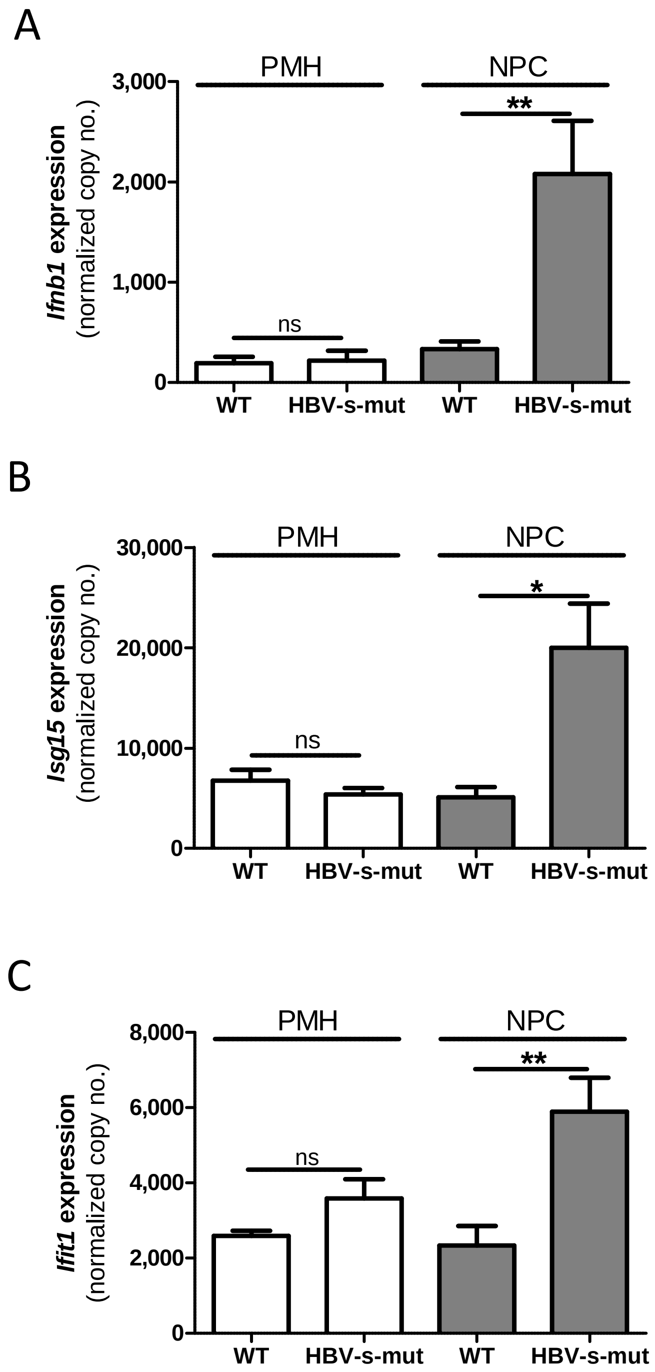

Supplementary figure 1. HBV-induced interferon responses are mediated by non-parenchymal liver cells (NPC). Primary murine hepatocytes (PMH) and non-parenchymal liver cell (NPC) fractions were isolated from two-month-old transgenic HBV (HBV-s-mut) mice and from their HBV-negative littermates (WT). RNA was extracted, and gene expression of interferon beta (*Ifnb1)* (A), interferon-stimulated gene 15 (*Isg15*) (B), and interferon-induced protein with tetratricopeptide repeats 1 (*Ifiti1*) (C) was determined by quantitative reverse transcription polymerase chain reaction (qRT-PCR). Copy numbers were normalized to 100,000 copies of glyceraldehyde 3-phosphate dehydrogenase (*Gapdh*) (mean±SEM). Group size n=3 animals; * p<0.05, ** p<0.01; ns, not significant.
